# DNA barcoding unravels contrasting evolutionary history of two widespread Asian tiger moth species during the Late Pleistocene

**DOI:** 10.1371/journal.pone.0194200

**Published:** 2018-04-04

**Authors:** Vitaly M. Spitsyn, Alexander V. Kondakov, Nikita I. Bolotov, Nhi Thi Pham, Mikhail Y. Gofarov, Ivan N. Bolotov

**Affiliations:** 1 Lab for Molecular Ecology and Phylogenetics, Northern Arctic Federal University, Arkhangelsk, Russian Federation; 2 Institute of Biogeography and Genetic Resources, Federal Center for Integrated Arctic Research, Russian Academy of Sciences, Arkhangelsk, Russian Federation; 3 Institute of Ecology and Biological Resources, Vietnam Academy of Science and Technology, Hanoi, Vietnam; 4 Graduate University of Science and Technology, Vietnam Academy of Science and Technology, Hanoi, Vietnam; Onderstepoort Veterinary Institute, SOUTH AFRICA

## Abstract

Populations of widespread pest insects in tropical areas are characterized by a complex evolutionary history, with overlapping natural and human-mediated dispersal events, sudden expansions, and bottlenecks. Here, we provide biogeographic reconstructions for two widespread pest species in the tiger moth genus *Creatonotos* (Lepidoptera: Erebidae: Arctiinae) based on the mitochondrial *cytochrome c oxidase subunit I* (*COI*) gene. The Asian *Creatonotos transiens* reveals shallow genetic divergence between distant populations that does not support its current intraspecific systematics with several local subspecies. In contrast, the more widespread *Creatonotos gangis* comprises at least three divergent subclades corresponding to certain geographic areas, i.e. Australia, Arabia + South Asia and Southeast Asia. With respect to our approximate Bayesian computation (ABC) model, the expansion of *Creatonotos gangis* into Australia is placed in the Late Pleistocene (~65–63 ka). This dating coincide with an approximate time of the earliest human migration into the continent (~65–54 ka) and the period of intervisibility between Timor and Australia (~65–62 ka). Our findings highlight that the drying Sunda and Sahul shelf areas likely support successful migrations of Asian taxa into Australia during the Pleistocene. The phylogeographic patterns discovered in this study can be used to improve the effectiveness of integrated pest control programs that is a task of substantial practical importance to a broad range of agricultural stakeholders.

## Introduction

Although the history of faunal exchange between mainland Southeast Asia and Australia attracts an exceptional attention of scientists since Alfred Russel Wallace [1], the patterns of this process are not entirely known. Typically, faunas of these regions are distinct, with intermediate forms ranging across New Guinea, Wallacea, Sundaland and the Philippines [2,3,4]. The molecular-based investigations revealed a higher level of endemism than previously recognized [3,4], e.g., 90% of Australian hawkmoths are endemic to the continent or to Australia, the Pacific Islands and the Papuan and Wallacean regions [4]. In contrast, several moth and butterfly species do not reveal deep genetic divergence between Australian and Southeast Asian populations, which indicates the possibility of long-distance dispersal events in these taxa during the Late Pleistocene [5,6,7,8]. The low sea levels through much of the Pleistocene support the exchange between the two continents via drying shelf areas creating Sundaland (mainland Southeast Asia with the Malay Peninsula and the Greater Sunda Archipelago) and Sahul (mainland Australia, Papua New Guinea and Tasmania) [9].

In the present study, we report the results of extensive phylogeographic analyses of *Creatonotos gangis* (Linnaeus, 1763) and *C*. *transiens* (Walker, 1855), two widespread pest tiger moth species (Erebidae: Arctiinae: Arctiini). The first species, *Creatonotos gangis*, is widely distributed in tropical and subtropical areas of the Old World, including the Middle East, South Asia, China, mainland Southeast Asia, Indonesia, the Philippines, New Guinea and Australia [10,11,12,13]. With respect to the morphology-based taxonomy, it appears to be a complex of closely related taxa ranged from the Middle East to Northern Australia [14]. Several species and subspecies-level taxa were described in this species complex [14,15]. The second species, *Creatonotos transiens*, also has a broad range, situated within Southeast Asia [12]. Both *Creatonotos gangis* and *C*. *transiens* are typical generalist species, which may inhabit a variety of environments, including agricultural lands and even urban areas (e.g., Delhi) [16,17]. The extreme polyphagy of larvae could explain a broad ecological niche in both species [18,19,20,21]. Interestingly, *Creatonotos gangis* appears to be highly adapted to rice, because the highest percentage of larval survival to pupation and the shortest larval development period occurred on this plant [21], hence it is an abundant species on rice fields [22]. This tiger moth species is also considered a possible pest of economically important plants such as sweet potato, turmeric, tea, soybean and maize [23,24]. Additionally, *Creatonotos gangis* may cause an extensive defoliation of pomegranate trees [25]. *Creatonotos transiens* is also a pest of many agricultural and forest plants, including the dragon tree, *Paulownia* spp. [26].

These moths are characterized by the dual mating system (both males and females produce pheromone) and the gigantic scent-bearing male coremata (androconia) [27,28]. The male coremata are pneumatically eversible organs composed of two pairs of tubes, up to 37 mm long, each covered by approximately 3000 long scales, with a giant epidermal (trichogen) gland cell at the base of each scale [27]. 7-Hydroxy-6,7-dihydro-5H-pyrrolizine-1-carboxaldehyde is the major volatile component of the coremata [29]. The size of coremata and the pheromone biosynthesis are directly controlled by the quantity of hostplant-derived pyrrolizidine alkaloids ingested by the larvae [29,30,18]. Despite the fact that *Creatonotos* species are both economically and scientifically important, their phylogeographic patterns are almost unknown, although several broad phylogenetic reconstructions contain sequences of certain *Creatonotos* species [31,32,33]. De Freina [34] provides a taxonomic revision of several African and Arabian *Creatonotos* taxa by means of a molecular approach.

Taking into account the above-mentioned broad ranges of our studied species, we assumed that they likely represent species complexes, each of which contains several cryptic species-level taxa. However, we found that the extensive distribution ranges of *Creatonotos gangis* and *C*. *transiens* do not reflect an ancient diversification pattern, and they could have been formed via multiple expansion events during the Late Pleistocene.

## Materials and methods

### Data collection, DNA extraction, PCR and sequencing

The barcode region of the fast evolving mitochondrial protein-coding *cytochrome c oxidase subunit I* (*COI*) gene was used in the present investigation. The sequence data set that combine our materials and published data comprises a total of 109 *Creatonotos* spp. sequences (S1 Table). For molecular analyses, we used 21 specimens of *Creatonotos gangis* and 20 specimens of *C*. *transiens* from the collection of the Russian Museum of Biodiversity Hotspots (RMBH), Federal Center for Integrated Arctic Research of the Russian Academy of Sciences, Arkhangelsk, Russian Federation. The total DNA was extracted from a single leg of each dry specimen using a standard phenol/chloroform procedure [35]. The standard primers C1-J-1718 and C1-N-2329R were used for the amplification of 612-bp-long barcode fragments of the *COI* gene [36]. The PCR mix contained approximately 200 ng of total cellular DNA, 10 pmol of each primer, 200 μmol of each dNTP, 2.5 μl of PCR buffer (with 10×2 mmol MgCl_2_), 0.8 units Taq DNA polymerase (SibEnzyme Ltd., Novosibirsk, Russia), and H_2_O added for a final volume of 25 μl. Thermocycling included one cycle at 95°C (4 min), followed by 36–38 cycles of 95°C (50 sec), 50°C (50 sec), and 72°C (50 sec) and a final extension at 72°C (5 min). Forward and reverse sequencing was performed on an automatic sequencer (ABI PRISM^®^ 3730, Applied Biosystems) using an ABI PRISM^®^ BigDye^™^ Terminator v. 3.1 reagent kit. Forty-one new *COI* sequences were obtained in this study, which were deposited in NCBI GenBank. Additionally, the published *COI* sequences of *Creatonotos* spp. were supplied by NCBI GenBank and BOLD IDS. All sequences were checked manually using a sequence alignment editor (BioEdit version 7.2.5 [37]). The alignment of sequences, which contained no indels, was performed using the ClustalW algorithm implemented in MEGA6 [38].

### Phylogenetic, phylogeographic and species delimitation analyses

For phylogenetic analyses, the sequence data set of *Creatonotos* spp. was collapsed into 61 unique *COI* haplotypes (648 bp in length) using an online FASTA sequence toolbox, FaBox v. 1.41 [39]. As an out-group, two additional *COI* haplotypes of *Arctia menetriesii* (Eversmann, 1846) and *A*. *tundrana* (Tshistjakov, 1990) were used (GenBank acc. nos. KM111174 and KM111175, respectively). The lacking sites were treated as missing data. The best models of sequence evolution as suggested the corrected Akaike Information Criterion (AICc) of MEGA6 [38] were as follows: 1^st^ codon of *COI*: TN93, 2^nd^ codon of *COI*: GTR, and 3^rd^ codon of *COI*: TN93+G (G = 1.1). Phylogenetic relationships were reconstructed based on Bayesian inference implemented in MrBayes v. 3.2.6 [40]. The analyses were performed using the following parameters: nchains = 4, nruns = 2, samplefreq = 1000, temp = 0.1; 10% of the sampled trees were discarded as burn-in (pre-convergence part). Runs were conducted for 25 million generations. Convergence of the MCMC chains to the stationary distribution was checked visually based on the plotted posterior estimates using a MCMC trace analysis tool (Tracer v1.6 [41]). Calculations were performed at the San Diego Supercomputer Center through the CIPRES Science Gateway [42].

The phylogeographic analyses of *Creatonotos* spp. sequences were performed based on a median joining network approach using Network v. 5.0.0.1 software with default settings [43]. The data set of 99 sequences across the extensive area from the Arabian Peninsula to Northern Australia was used (S1 Table). To remove missing sites due to different length of available sequences, all sequences were cut in accordance with the minimal sequence length leaving the data set of 456 bp long. Several shorter sequences were removed from the data set.

To test the hypothesis that *Creatonotos gangis* and *C*. *transiens* may represent complexes of closely related species-level taxa [14,15,34], we applied a species delimitation approach by using the Bayesian Poisson Tree Process (PTP) model [44]. Using the fifty-percent majority-rule Bayesian consensus tree (see above), two runs of 100,000 generations were executed with the first 10% discarded as a burn-in based on the convergence of log-likelihood values (S1 Fig). The two out-group taxa were retained for the analysis, because the number of in-group taxa was low that may biased the delimitation results. Additionally, we performed a standard barcoding gap analysis based on uncorrected *COI p*-distances [45,46] in order to supplement the PTP modeling results.

### Population genetic analysis and approximate Bayesian computation

Population genetic diversity indexes (haplotype and nucleotide diversity), Tajima’s *D*-test and Fu’s *F*-test statistics and the mismatch distribution under a spatial expansion model were calculated using Arlequin v. 3.5.1.2 software to estimate the demographic histories of sampled populations [47,48]. A total sequence sample was subdivided in accordance with geographic areas and taxa: (i) *Creatonotos gangis*, Arabia and South Asia (*N* = 20); (ii) *C*. *gangis*, mainland Southeast Asia (*N* = 20); (iii) *C*. *gangis*, Eurasia (*N* = 40); (iv) *C*. *gangis*, Australia (*N* = 15); (vi) *C*. *gangis*, all available sequences (Eurasia + Lesser Sundas + Australia, *N* = 57); and (vii) *C*. *transiens*, Eurasia (*N* = 39). Nucleotide differences (uncorrected *p*-distance, %) between sequence groups with standard error values based on 1000 bootstrap replications were calculated in MEGA6 [38].

The time since population expansion (*t*, generations) was calculated using the equation:

t=τ/2μ; (1)

where τ is a moment estimator that represents a unit of mutational time, inferred from the mode of mismatch distribution, and μ is a mutation rate (%) per site per generation [7,49,50,51]. The most appropriate external *COI* mutation rate of 1.77×10^−8^ substitutions/site/year was applied, which is based on the mid-Aegean trench calibration using six genera of darkling beetles (Coleoptera: Tenebrionidae) [52].

Finally, the two integrative biogeographic scenarios concerning the possible origin of *Creatonotos gangis* populations were designed (S5 Table). Both scenarios assume that Arabian–South Asian and Australian populations independently derived from Southeast Asian population, but the scenario 1-CG suggests more ancient divergence times (S5 Table). These demographic scenarios were simulated for comparison using an approximate Bayesian computation (ABC) approach with DIYABC v. 2.1.0 software [53,54]. The primary sequence data set included three samples of *Creatonotos gangis* sequences: (i) Southeast Asia (*N* = 20); (ii) Arabia and South Asia (*N* = 20); and (iii) Australia (*N* = 15). Prior settings of the ABC analyses are presented in S5 Table. The HKY+G model was likely the best model of evolution for our data set in accordance with the AICc of MEGA6 [38]. A total of 2×10^6^ simulated data sets were calculated. Pre-evaluations of model-prior combinations in ABC inference revealed that the prior settings were correctly assigned (S2 Fig; [53]). The model checking indicated that posterior combination under each scenario corresponded well with a target data set (S2 Fig), which confirmed the ‘goodness-of-fit’ of the models [53].

## Results

### Phylogenetic reconstruction, phylogeography and species delimitation

**Fig 1:**
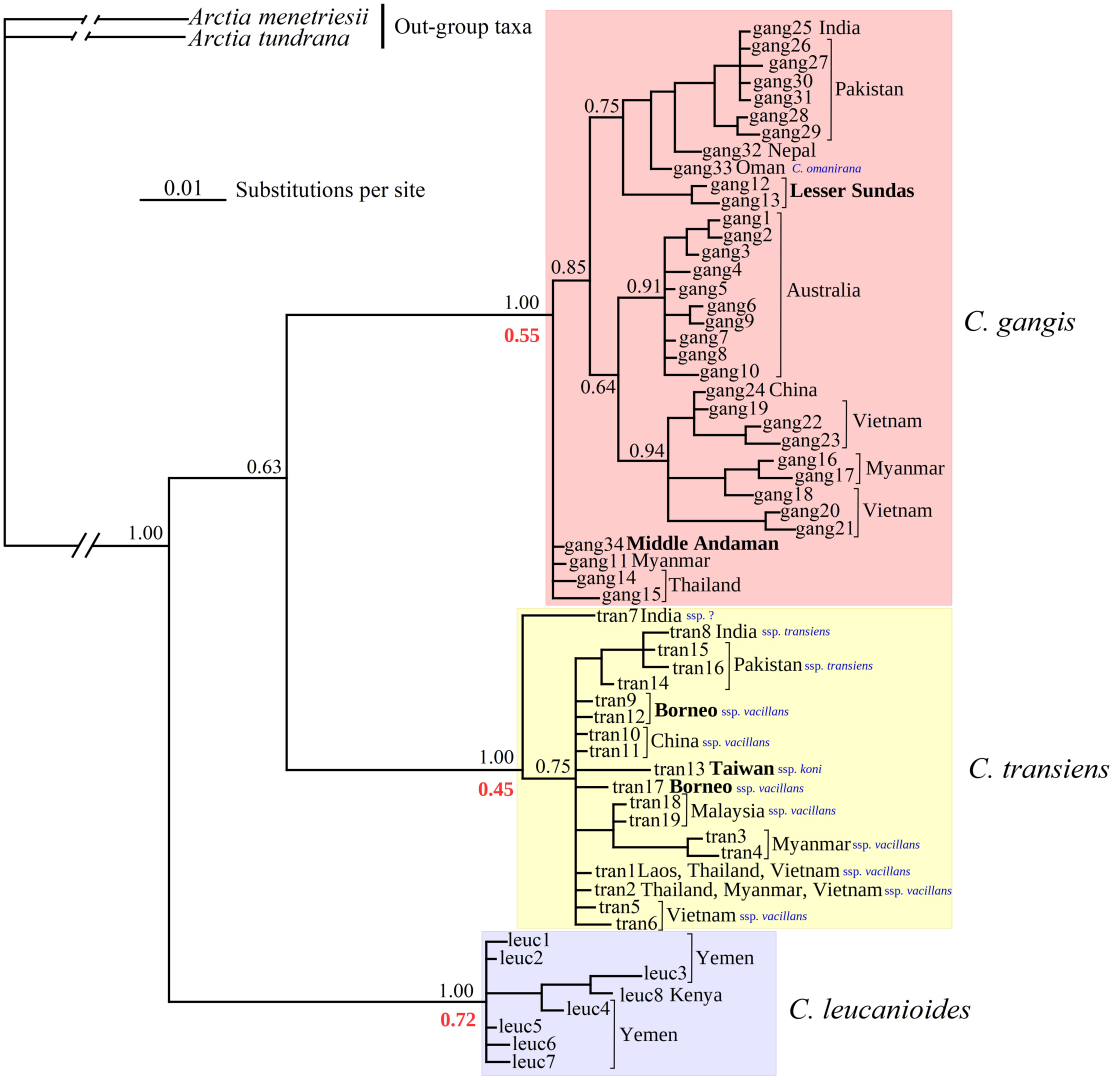
Fifty-percent majority-rule consensus phylogenetic tree of *Creatonotos* spp. recovered from Bayesian inference analysis of an alignment comprising 61 *COI* haplotypes of *Creatonotos* spp. and two haplotypes of the out-group taxa (*Arctia menetriesii* and *A*. *tundrana*). The island localities are in bold. Black numbers near branches are Bayesian posterior probabilities (BPP). The red number near each primary clade is the probability of each species-level MOTU based on the highest Bayesian supported solution of the PTP model.

The Bayesian phylogeny of *Creatonotos* spp. based on the 61 *COI* haplotypes reveals the three species-level clades with high Bayesian posterior probabilities (BPP = 1.00) (Fig 1 and S1 Table). The haplotype of *Creatonotos omanirana* De Freina, 2007 is located within haplotypes of *C*. *gangis* from South Asia (India, Pakistan and Nepal) and the Lesser Sunda Archipelago (Flores). Among the taxa under discussion, *Creatonotos gangis* shows the strongest spatial structure of genetic diversity. There are three subclades of the *Creatonotos gangis* haplotypes with moderate support: Southeast Asia (BPP = 0.94), Australia (BPP = 0.91), and South Asia + the Arabian Peninsula + Lesser Sundas (BPP = 0.75). The Bayesian modeling did not resolve the position of several haplotypes from Myanmar, Thailand and Middle Andaman (gang11, gang14, gang15 and gang34) although the median-joining network analysis, which was calculated on the basis of the short length sequence data set, placed these haplotypes within the Southeast Asian haplogroup (see below). In contrast, *Creatonotos transiens* does not reveal such a spatial structure, with the only divergent haplotype from India (Western Ghats). All of the other haplotypes (Southeast Asia, South Asia, Taiwan and Borneo) are sampled into a single polytomic clade with moderate support (BPP = 0.75) (Fig 1).

**Fig 2:**
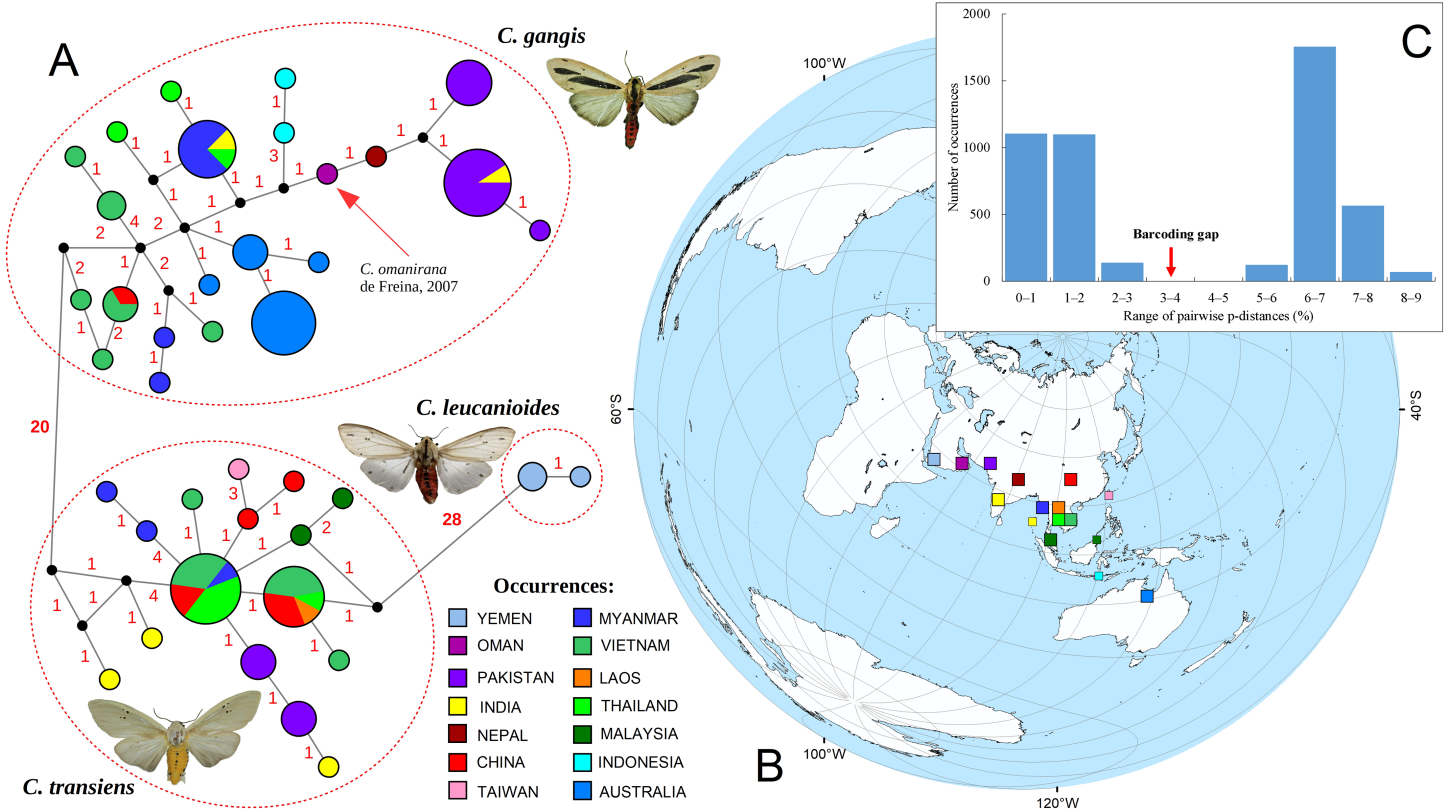
Phylogeography of *Creatonotos* spp. (**A**) Median-joining network of *COI* sequences (see S1 Table for details). Photos (male specimens): *C*. *gangis* [Indonesia, Flores Island, voucher no. Sph0595] and *C*. *transiens* [Thailand, near Tham Lod Cave, voucher no. Sph0624] by Vitaly M. Spitsyn and *C*. *leucanioides* [Tanzania] by Roy Goff (with his permission; www.africanmoths.com). (**B**) Map of approximate collection localities of the specimens in accordance with the respective countries (color squares). Small squares indicate island sites. The map was created using ESRI ArcGIS 10 software (www.esri.com/arcgis); the base of the map was created with ESRI Data and Maps. (**C**) Frequency histogram of the barcoding gap analysis.

The median-joining network reflects the same pattern, with three groups of closely related haplotypes (1–4 mutational steps) corresponding to the three species, which are separated by 20–28 nucleotide substitutions (Fig 2). The sequences of *Creatonotos transiens* show the ‘star-like’ network, which includes the two most frequently recorded haplotypes, which have broad range across Southeast Asia, with supplement of many local singletons. In contrast, the network of *Creatonotos gangis* is spatially structured, with several distant haplogroups ranged in Southeast Asia, Australia, and Arabia/South Asia (Fig 2). The haplotype of *Creatonotos omanirana* is located between haplotypes from Southeast Asia (Myanmar, Thailand, Middle Andaman and Flores) and Nepal. The highest Bayesian supported solution of the PTP model also support the three species-level MOTUs (Fig 1 and S1 Fig), but with moderate probabilities (0.45–0.72). The results of the barcoding gap analysis show a well-defined 3% species-level threshold (Fig 2). The mean genetic divergences between *Creatonotos gangis* populations are ≤1.78%, whereas the minimum interspecific distance of *Creatonotos* spp. is 6.41% (S2 Table).

### Historical demography and divergence time estimates

With respect to the approach of Grant & Bowen [55], the Australian and Arabian-South Asian populations of *Creatonotos gangis* differ by large haplotype diversity (h ranges from 0.54 to 0.66) and small nucleotide diversity (π ranges from 0.2 to 0.4%) (S3 Table). All of the other samples of *Creatonotos gangis* and the sample of *C*. *transiens* reveal large haplotype diversity (h >0.85) and large nucleotide diversity (π >0.5%). The values of Fu’s *FS* and Tajima’s *D* tests indicate no deviation from mutation-drift equilibrium for the samples of *Creatonotos gangis*, whereas the *C*. *transiens* sample has significant negative values of both statistics revealing a possible historic demographic expansion (S3 Table).

**Fig 3:**
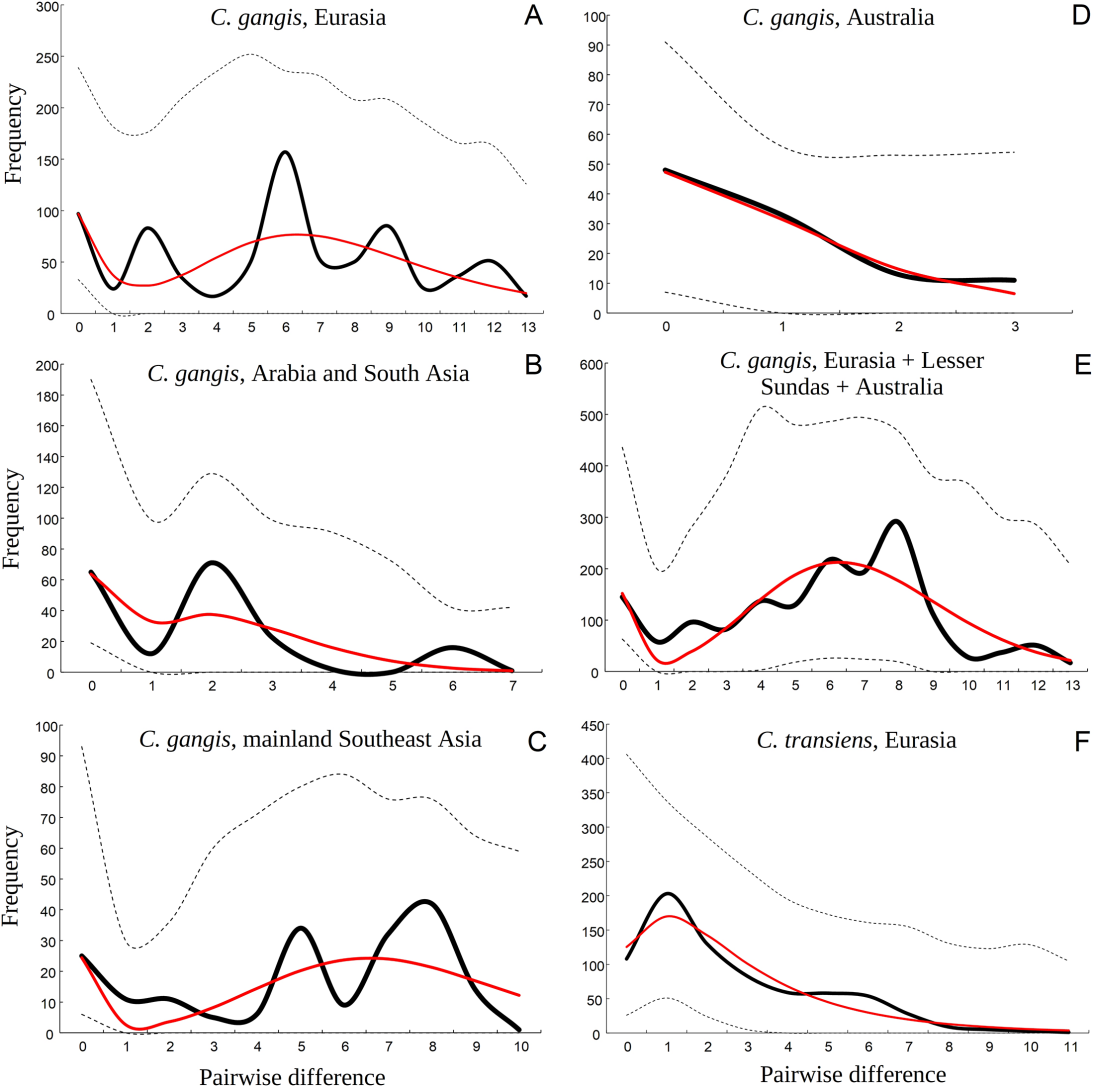
Mismatch distributions of *Creatonotos* spp. samples based on the mitochondrial *COI* gene. Solid black lines indicate observed distribution, and solid red lines represent simulated distribution under a spatial expansion model. Dashed lines represent lower and upper confidence intervals (*p* = 0.01). (**A**) *C*. *gangis*, Eurasia (*N* = 40 sequences; Raggedness *P* = 0.342; Model (SSD) *P* = 0.317). (**B**) *C*. *gangis*, Arabia and South Asia (*N* = 20 sequences; Raggedness *P* = 0.264; Model (SSD) *P* = 0.273). (**C**) *C*. *gangis*, mainland Southeast Asia (*N* = 20 sequences; Raggedness *P* = 0.089; Model (SSD) *P* = 0.331). (**D**) *C*. *gangis*, Australia (*N* = 15 sequences; Raggedness *P* = 0.910; Model (SSD) *P* = 0.736). (**E**) *C*. *gangis*, the entire range (*N* = 57 sequences; Raggedness *P* = 0.715; Model (SSD) *P* = 0.557). (**F**) *C*. *transiens*, Eurasia (*N* = 39 sequences; Raggedness *P* = 0.671; Model (SSD) *P* = 0.596).

The mismatch distribution analysis of the Southeast Asian and Arabian-South Asian populations of *Creatonotos gangis* resulted in a multimodal distribution with three peaks at 0, 5 and 8 bp (Southeast Asia) and at 0, 2 and 6 bp (Arabia and South Asia) (Fig 3). The general samples of *Creatonotos gangis* (Eurasia and all available sequences) also reveal multiple peaks, whereas the Australian population has a unimodal distribution with the maximum value at 0 bp. The sample of *Creatonotos transiens* from Eurasia shows a bimodal distribution with peaks at 1 and 6 bp. The Australian population of *Creatonotos gangis* and the sample of *C*. *transiens* from Eurasia reveal the lowest values of the parameter τ, which reflects the time since expansion, while all of the other samples of *C*. *gangis* return much larger moment estimator values (S3 Table). According to the ‘mid-Aegean’ mutation rate of 1.77×10^−8^ s/s/y (substitutions/site/year), the mean time since expansion of the Arabian-South Asian population of *Creatonotos gangis* was 640 ka (thousands of years) and that of the Australian population was 67 ka (S3 Table). The time since expansion of *Creatonotos transiens* population was 196 ka.

**Fig 4:**
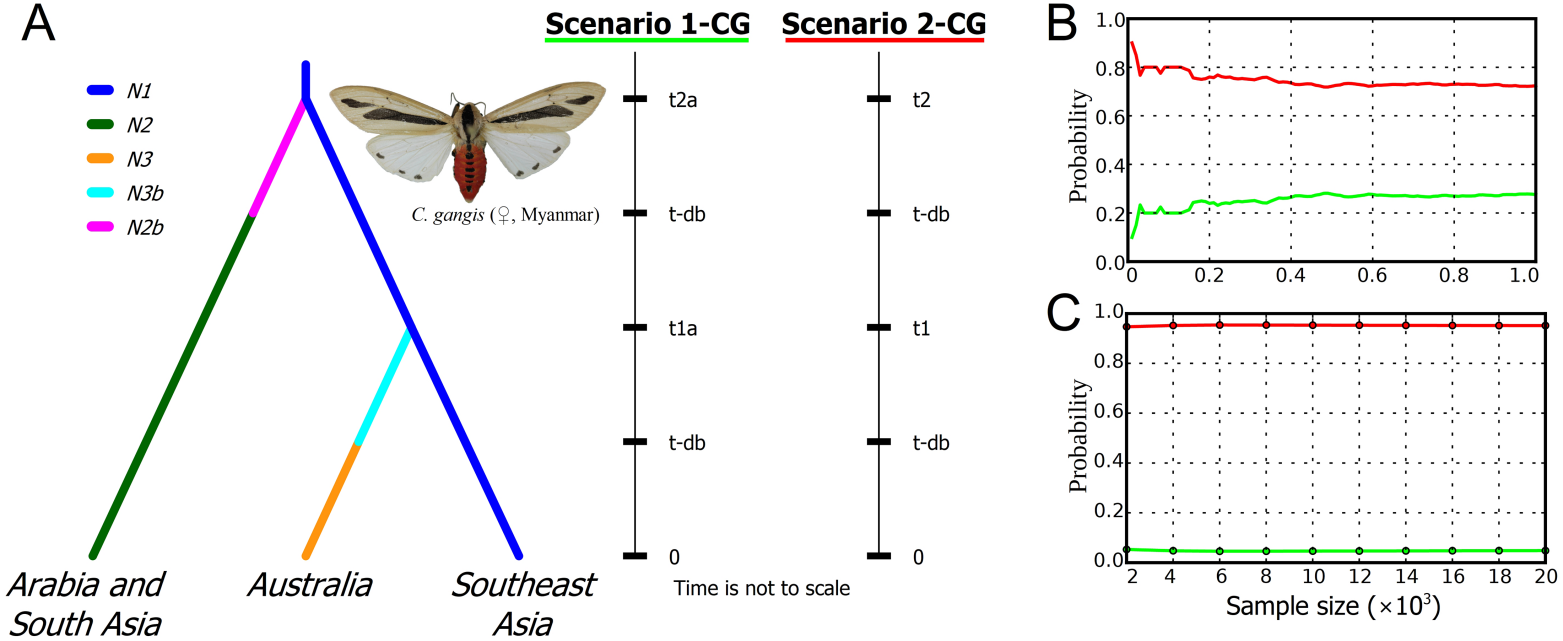
ABC modeling of origin of the *Creatonotos gangis* populations. (**A**) Biogeographic scenarios that were tested under an ABC framework using *COI* gene sequences. Southeast Asian population: samples from Myanmar, Vietnam, Thailand, and South China, Arabian–South Asian population: samples from Oman, Pakistan, India, and Nepal, and Australian population. Effective population size: *N*_1_ –Southeast Asian population; *N*_2_ –Arabian–South Asian population; *N*_3_ –Australian population; *N*_2b_ and *N*_3b_ –hypothetical founder population for Arabian–South Asian and Australian populations, respectively. Time intervals: *t*_1a_ and *t*_1_ –time of the primary split between populations *Pop1* and *Pop2* under scenarios 1-CG (before the mid-Pleistocene) and 2-CG (since the mid-Pleistocene), respectively; *t*_2a_ and *t*_2_ –time of split between Southeast Asian and Australian populations under scenarios 1-CG (before the Late Pleistocene) and 2-CG (during the Late Pleistocene), respectively; *t*-*db*–period of low effective population size *N*_2b_ and *N*_3b_ since colonization of the Arabian–South Asian Region and Australia. Prior settings are presented in S5 Table. Evaluating the confidence in scenario choice using the direct (**B**) and linear regression (**C**) approaches.

**Fig 5:**
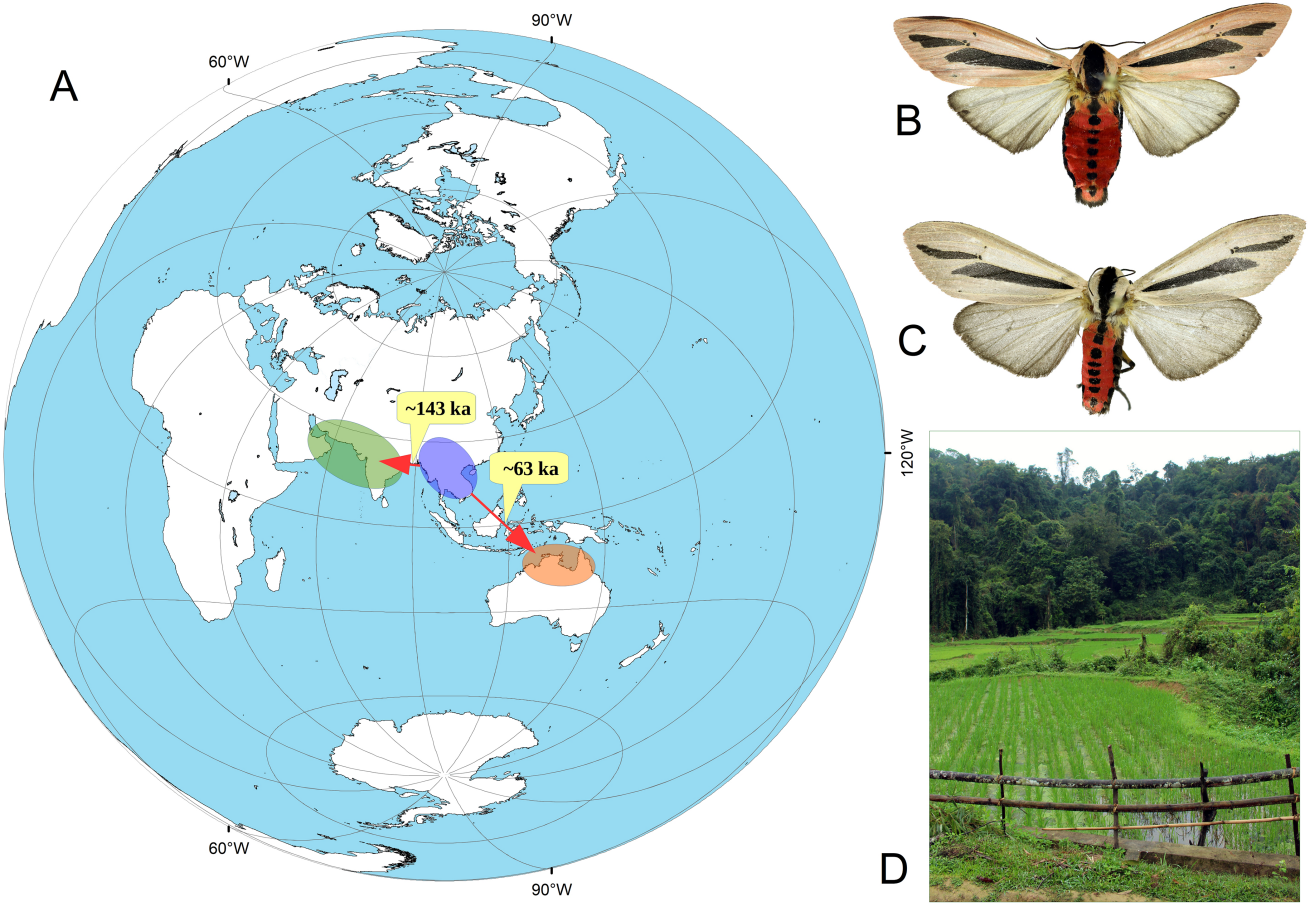
Simplified summary of expansion routes inferred across populations of *Creatonotos gangis*, and examples of male specimens and habitat of Southeast Asian population. (**A**) Map of expansion routes. Color circles indicate an approximate range of each population: Southeast Asian population (blue), Arabian–South Asian population (green), and Australian population (orange). Red arrows indicate the directions of expansion routes. The numbers near arrows show the mean age of putative expansion events (in thousands of years) obtained from the ABC model. The map was created using ESRI ArcGIS 10 software (www.esri.com/arcgis); the base of the map was created with ESRI Data and Maps. (**B**) Male specimen, Maehongson, Thailand. (**C**) Male specimen, Kachin, Myanmar. (**D**) Paddy field, a typical habitat of the species, Thanh Hoa Province, Vietnam. (Photos: Vitaly M. Spitsyn).

The results of the ABC modeling reveal that the scenario 2-CG, which proposed more recent dispersal events into South Asia, Arabia and Australia, was assigned as the most likely scenario with a high posterior probability (Fig 4). The posterior predictive error rate, which was calculated over 1000 data sets using the direct approach, was 0.151. Type I and Type II errors for the choice of scenario 2-CG in accordance with the direct approach were 0.234 and 0.144, respectively. Modeling results, obtained under the scenario 2-CG, reveal the most recent dating of divergence times for the splits between populations of *Creatonotos gangis* (S4 Table). In particular, the Arabian-South Asian population most likely derived from the Southeast Asian population before the Late Pleistocene (mean age 143.0 ka, median age 109.0 ka, 95% CI 55.8–359.0 ka), while the Australian population most likely originated in the Late Pleistocene (mean age 62.6 ka, median age 65.1 ka, 95% CI 19.3–96.9 ka) (Fig 5). Our model predicts a relatively slow substitution rate in populations of *Creatonotos gangis* (mean rate 1.52×10^−8^ s/s/y, median rate 1.35×10^−8^ s/s/y, 95% CI 1.03×10^−8^–2.58×10^−8^ s/s/y).

## Discussion

### Biogeography of *Creatonotos* spp. and possible Late Pleistocene expansions of Asian taxa into Sahul

In general, the results of our phylogenetic and population genetic analyses support the conclusion that both *Creatonotos gangis* and *C*. *transiens* are widespread polymorphic species. *Creatonotos gangis* comprises at least three divergent intraspecific subclades corresponding to certain geographic areas, i.e., Australia, Arabia + South Asia and Southeast Asia. This pattern may reflect ancient splitting events. Based on the approach of Grant & Bowen [55], large haplotype diversity together with small nucleotide diversity in Arabian-South Asian and Australian samples (h >0.5, π <0.5%) may indicate a population bottleneck since a founder event followed by rapid population growth and accumulation of mutations. According to this scenario, Southeast Asia appears to be the most probable ancestral area of *Creatonotos gangis*, from which this species has colonized other areas during the Late Pleistocene. The ABC modeling suggests that the Australian clade likely separated from the Southeast Asian populations at ~65–63 ka (median and mean estimations of the 2-CG scenario, respectively). This dating roughly coincided with that of the earliest human expansion crossing the sea-level-altered Indonesian Archipelago and arrive into Sahul at ~65–54 ka [56,57,58,59,60]. This human expansion was largely driven by orbital-scale global climate swings [58] that could have been accelerated a long-distance dispersal of *Creatonotos gangis* and other Asian insect taxa. The inter-island visibility modeling of Bergström et al. [56] provides an evidence for intervisibility between Timor and Australia at approximately 65–62 ka, which corresponds to the time of *Creatonotos gangis* expansion inferred from our ABC model. In contrast, specimens from the Lesser Sundas are not sister to the Australian lineage (Figs 1 and 2) but show affinities to the Arabian-South Asian subclade that may indicate a secondary, more recent expansion from the latter region into Wallacea. The Andaman Islands were likely recently colonized by the representatives of *Creatonotos gangis* from Southeast Asia (Figs 1 and 2).

These patterns differ from those discovered in many other insect taxa. For example, *Lampides boeticus* (Linnaeus, 1767), one of the most widely distributed butterfly species in the world, expands into Australia several times from populations of Sundaland and Wallacea [7]. Additionally, Australian populations of this butterfly have very low mtDNA divergence from corresponding Sundaic and Wallacean populations, which suggests an invasion since the Last Glacial Maximum. The same phylogeographic pattern with a small genetic divergence between Southeast Asian and Australian populations was observed in other widespread species, e.g., *Zizina otis* (Fabricius, 1787), *Danaus chrysippus* (Linnaeus, 1758), and *Asota caricae* (Fabricius, 1775) [5,6,8], and even in the migratory locust *Locusta migratoria* (Linnaeus, 1758) [61].

The other studied species, *Creatonotos transiens*, shows relatively shallow genetic divergence between populations across the entire distribution range, which suggests a possible sudden population expansion during the Late Pleistocene. However, in accordance with the approach of Grant & Bowen [55], large haplotype diversity together with large nucleotide diversity in the sample (h = 0.85, π = 0.6%) may indicate large stable population with long evolutionary history or secondary contact between differentiated lineages. Similar shallow genetic divergence was recorded in *Arctia plantaginis* (Linnaeus, 1758), another tiger moth species, within its range from Eurasia to North America [62]. The Polar tiger moth *Arctia tundrana* (Tshistjakov, 1990) shares closely related haplotypes across the Eurasian Arctic at the distance of ~5,000 km [63]. Rönkä et al. [64] show that many taxa within the widespread *Arctia caja* (Linnaeus, 1758) species complex form a single cluster with high support and very little genetic difference. Similar patterns were discovered in many moth and butterfly species from other families [5,6,8,65,66,67]. These findings highlight the role of long-distance dispersal events connecting distinct populations by gene flows, which leads to the lack of significant genetic divergences between individuals from geographically remote areas. We suggest that such species can exist as the huge metapopulations with broad ranges, which may cover extensive areas within one or even two continents, and hence these taxa do not reveal clear spatial differentiation on geographic races (subspecies).

### Putative taxonomic implications

De Freina [15] described *Creatonotos omanirana* as a new species, which is closely related to *C*. *gangis*. However, our wide-scale phylogeny reveals that this taxon is rather a variation of *Creatonotos gangis*, because the available *COI* sequence of *C*. *omanirana* falls within the *C*. *gangis* clade and the morphological differences between these taxa are small [15,34] (Figs 1 and 2). Our phylogenetic reconstructions also suggest that the *Creatonotos gangis* clade may include several subspecies-level taxa, e.g., from Australia and from Lesser Sundas, but a broader sampling is needed to access the fully resolved phylogeny with well-supported subspecies-level clades. Additionally, *Creatonotos fasciatus* Candèze, 1927, a prospective sister species of *C*. *gangis* [14], has been lacking in our samples. This poorly known species is in need of future molecular investigations.

As for the intraspecific phylogeny of *Creatonotos transiens*, we found no clear support for the two subspecies that were considered valid by Dubatolov and Holloway [68]: *C*. *transiens koni* Miyake, 1909 from Taiwan and Japan, and *C*. *transiens vacillans* (Walker, 1855) from China, Indo-China, the Malay Peninsula, Sumatra and Borneo. Several other subspecies [68] are beyond the framework of the present study, and they are still waiting for molecular investigations. In contrast, our sample includes a divergent *Creatonotos transiens* sequence from the Western Ghats, in which a separate subspecies may be present, but it needs to be confirmed in the future.

In summary, our new molecular-based findings could be used as a supplement for future taxonomic revisions of *Creatonotos gangis* and *C*. *transiens* because both these species include several divergent intraspecific mtDNA lineages that may be considered separate evolutionary significant units.

## Supporting information

S1 Fig Bayesian species delineation using the Poisson Tree Process (PTP) model based on the distribution of nucleotide substitutions in the *COI* Bayesian phylogeny of *Creatonotos* spp. Terminal branches in blue indicate lineages that stand as separate Molecular Operational Taxonomic Units (MOTUs) and the clades in red are lumped into a single MOTU. Numbers near branches are support values of each MOTU based on the PTP model.
(PDF)

S2 Fig Test results of biogeographical scenarios 1-CG and 2-CG concerning origin of the *Creatonotos gangis* populations under an ABC framework using the *COI* gene sequences. (**a**) Pre-evaluation of prior combinations of scenarios. (**b)**-(**c**). Model checking to measure a mismatch between the parameters of posterior combination and observed data sets in scenarios 1- CG (**b**) and 2- CG (**c**). Scenarios are illustrated in Fig 4. A description of basic assumptions with prior settings for each scenario is presented in S5 Table.
(PDF)

S1 Table List of the mitochondrial *COI* sequences of *Creatonotos* spp. examined in the present study. (PDF)

S2 Table Genetic divergences (mean uncorrected *p*-distance ± standard error estimations, %) between populations and taxa of *Creatonotos* spp. based on the mitochondrial *COI* gene fragment. (PDF)

S3 Table Molecular diversity indexes and population expansion test statistics of *Creatonotos* spp. samples based on the *COI* sequences. (PDF)

S4 Table Model parameters estimated from posterior distribution of the scenario 2-CG concerning origin of *Creatonotos gangis* populations within the ABC framework. (PDF)

S5 Table Prior assumptions and settings of the two biogeographical scenarios of the origin of *Creatonotos gangis* populations, which were tested under an ABC framework. (PDF)
