# Supplementary material for: DNA barcoding unravels contrasting evolutionary history of two widespread Asian tiger moth species during the Late Pleistocene
Source: PLoS One. 2018 Apr 4;13(4):e0194200. doi: 10.1371/journal.pone.0194200 (PMC5884489; doi:10.1371/journal.pone.0194200)
Supplement: S1 Fig — Terminal branches in blue indicate lineages that stand as separate Molecular Operational Taxonomic Units (MOTUs) and the clades in red are lumped into a single MOTU. Numbers near branches are support values of each MOTU based on the PTP model. (PDF) [file pone.0194200.s001.pdf]

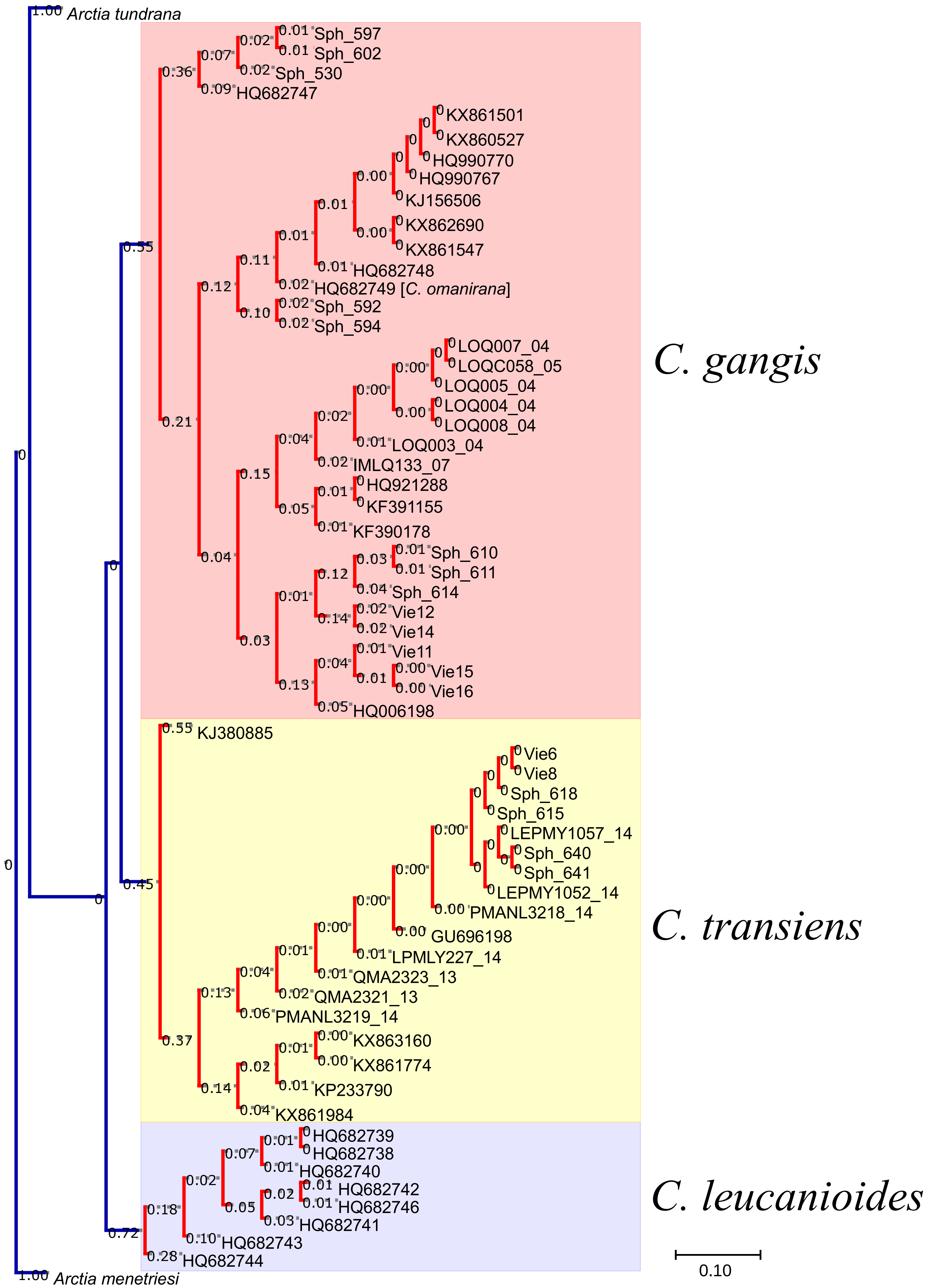

S1 Figure. Bayesian species delineation using the Poisson Tree Process (PTP) model based on the distribution of nucleotide substitutions in the COI Bayesian phylogeny of *Creatonotos* spp. Terminal branches in blue indicate lineages that stand as separate Molecular Operational Taxonomic Units (MOTUs) and the clades in red are lumped into a single MOTU. Numbers near branches are support values of each MOTU based on the PTP model.
