# Supplementary material for: DNA barcoding unravels contrasting evolutionary history of two widespread Asian tiger moth species during the Late Pleistocene
Source: PLoS One. 2018 Apr 4;13(4):e0194200. doi: 10.1371/journal.pone.0194200 (PMC5884489; doi:10.1371/journal.pone.0194200)
Supplement: S2 Fig — (a) Pre-evaluation of prior combinations of scenarios. (b)-(c). Model checking to measure a mismatch between the parameters of posterior combination and observed data sets in scenarios 1- CG (b) and 2- CG (c). Scenarios are illustrated in Fig 4. A description of basic assumptions with prior settings for each scenario is presented in S5 Table. (PDF) [file pone.0194200.s002.pdf]

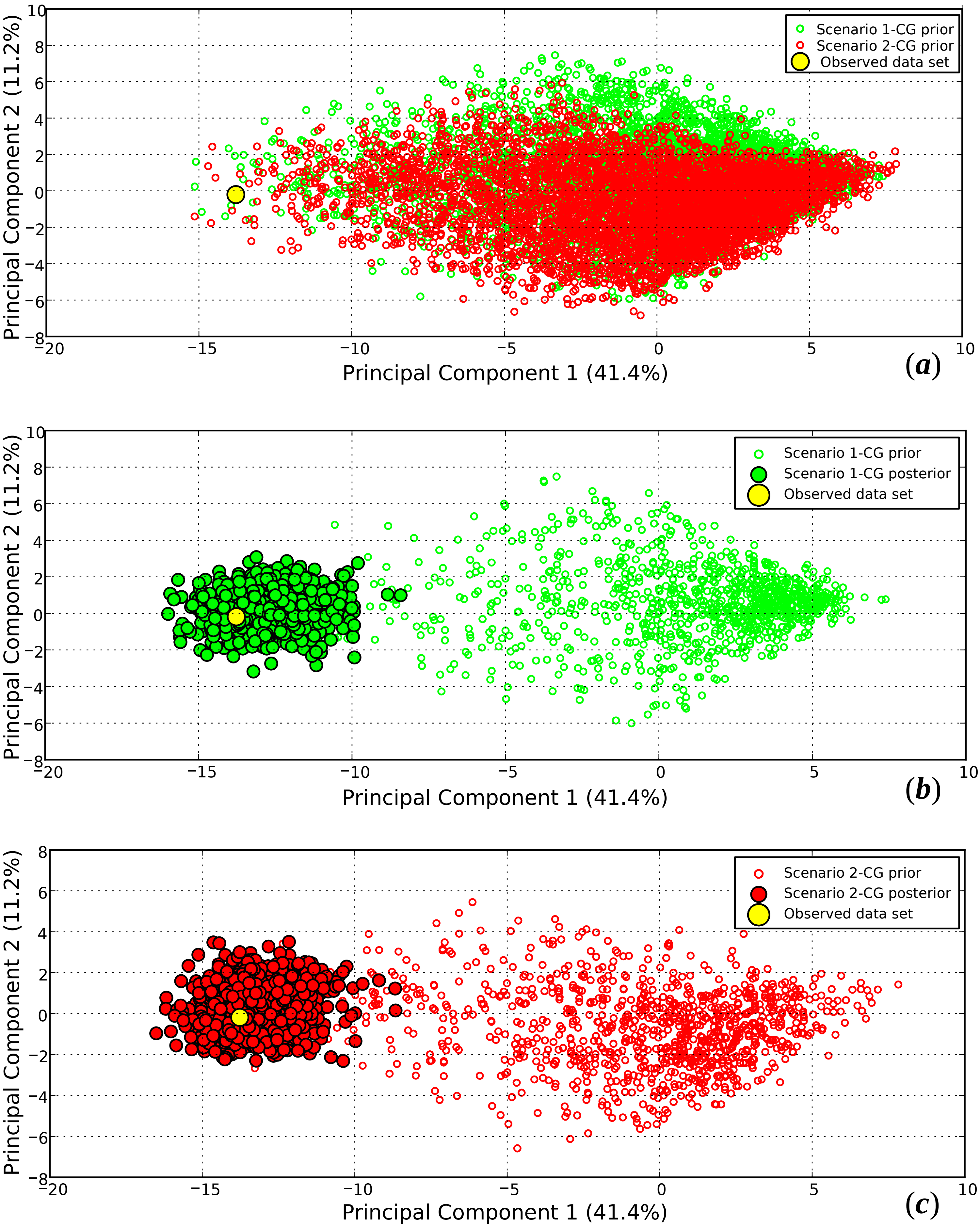

S2 Figure. Test results of biogeographical scenarios 1-CG and 2-CG concerning origin of the *Creatonotos gangis* populations under an ABC framework using the COI gene sequences. (**a**) Pre-evaluation of prior combinations of scenarios. (**b**)-(**c**). Model checking to measure a mismatch between the parameters of posterior combination and observed data sets in scenarios 1- CG (**b**) and 2- CG (**c**). Scenarios are illustrated in Figure 4. A description of basic assumptions with prior settings for each scenario is presented in Supplementary Table 2.
