# Supplementary material for: DNA barcoding unravels contrasting evolutionary history of two widespread Asian tiger moth species during the Late Pleistocene
Source: PLoS One. 2018 Apr 4;13(4):e0194200. doi: 10.1371/journal.pone.0194200 (PMC5884489; doi:10.1371/journal.pone.0194200)
Supplement: S1 Table — (PDF) [file pone.0194200.s003.pdf]

S1 Table. List of the mitochondrial COI sequences of *Creatonotos* spp. examined in the present study*

Species	Haplotype Code	GenBank / BOLD IDS acc. no.	Voucher no.	Locality	Data source***
C. gangis	gang15	KY683804	Sph602	Thailand: Loei Province	Present study
C. gangis	gang11	KY683803	Sph600	Thailand: Maehongson	Present study
C. gangis	gang14	KY683802	Sph597	Thailand: Maehongson	Present study
C. gangis	gang18	KY683812	Sph614	Vietnam: Quang Nam Province	Present study
C. gangis	gang19	KY683833	Vie11	Vietnam: Thanh Hoa Province	Present study
C. gangis	gang20	KY683834	Vie12	Vietnam: Thanh Hoa Province	Present study
C. gangis	gang20	KY683835	Vie13	Vietnam: Thanh Hoa Province	Present study
C. gangis	gang21	KY683836	Vie14	Vietnam: Thanh Hoa Province	Present study
C. gangis	gang22	KY683837	Vie15	Vietnam: Thanh Hoa Province	Present study
C. gangis	gang23	KY683838	Vie16	Vietnam: Thanh Hoa Province	Present study
C. gangis	gang19	KY683839	Vie17	Vietnam: Vinh Phuc Province	Present study
C. gangis	gang11	KY683799	Sph530	Myanmar: Kachin State	Present study
C. gangis	gang11	KY683805	Sph603	Myanmar: Kachin State	Present study
C. gangis	gang11	KY683806	Sph604	Myanmar: Kachin State	Present study
C. gangis	gang11	KY683807	Sph605	Myanmar: Kachin State	Present study
C. gangis	gang11	KY683808	Sph 606	Myanmar: Kachin State	Present study
C. gangis	gang11	KY683809	Sph 607	Myanmar: Kachin State	Present study
C. gangis	gang16	KY683810	Sph610	Myanmar: Mandalay Region	Present study
C. gangis	gang17	KY683811	Sph611	Myanmar: Mandalay Region	Present study
C. gangis	gang24	HQ006198	RZ30	China: Hong Kong	[69]
C. gangis	gang12	KY683800	Sph592	Lesser Sundas: West Flores	Present study
C. gangis	gang13	KY683801	Sph594	Lesser Sundas: West Flores	Present study
C. gangis	gang25	KJ156506	ERI-RH-M004	India	GenBank
C. gangis	gang34	HQ682747	JDF0012	India: Andaman Islands, Middle Andaman Island	[34]
C. gangis	gang32	HQ682748	JDF0010	Nepal: Trisuli valley	[34]
C. gangis	gang26	KX863293	NIBGE MOT-01788	Pakistan: Kashmir	GenBank
C. gangis	gang30	KX861501	NIBGE MOT-02893	Pakistan: Khyber Pakhtunkhwa	GenBank
C. gangis	gang31	KX860527	NIBGE MOT-02894	Pakistan: Khyber Pakhtunkhwa	GenBank
C. gangis	gang26	KX861886	NIBGE MOT-02889	Pakistan: Punjab	GenBank
C. gangis	gang26	KX860950	NIBGE MOT-02890	Pakistan: Punjab	GenBank
C. gangis	gang26	KX863253	NIBGE MOT-02891	Pakistan: Punjab	GenBank
C. gangis	gang26	KX861026	NIBGE MOT-02892	Pakistan: Punjab	GenBank
C. gangis	gang26	HQ990767	NIBGE MOT-00044	Pakistan: Punjab	GenBank
C. gangis	gang26	HQ990768	NIBGE MOT-00045	Pakistan: Punjab	GenBank
C. gangis	gang26	HQ990769	NIBGE MOT-00046	Pakistan: Punjab	GenBank
C. gangis	gang27	HQ990770	NIBGE MOT-00047	Pakistan: Punjab	GenBank
C. gangis	gang29	KX861205	NIBGE MOT-01500	Pakistan: Punjab	GenBank
C. gangis	gang28	KX860740	NIBGE MOT-01621	Pakistan: Punjab	GenBank

Species	Haplotype Code	GenBank / BOLD IDS acc. no.	Voucher no.	Locality	Data source***
C. gangis	gang28	KX862690	NIBGE MOT-01497	Pakistan: Sind	GenBank
C. gangis	gang28	KX861833	NIBGE MOT-01498	Pakistan: Sind	GenBank
C. gangis	gang29	KX861547	NIBGE MOT-01499	Pakistan: Sind	GenBank
C. gangis (=C. omanirana)	gang33	HQ682749	JDF0011	Oman: Jabal Akhdar-No-Auslaeufer, Oase Nakhl	[34]
C. gangis	gang1	HQ921288	10ANIC-01171	Australia: Northern Territory	[70]
C. gangis	gang2	KF391155	10ANIC-01172	Australia: Northern Territory	[70]
C. gangis	gang3	KF390178	10ANIC-01173	Australia: Northern Territory	[70]
C. gangis	gang4	IMLQ133-07	IM06-0446	Australia: Queensland	BOLD IDS
C. gangis	gang4	IMLQ185-07	IM06-0756	Australia: Queensland	BOLD IDS
C. gangis	gang4	IMLQ979-08	IM08-0367	Australia: Queensland	BOLD IDS
C. gangis	gang4	IMLQ988-08	IM08-0378	Australia: Queensland	BOLD IDS
C. gangis	gang4	IMLR943-11	IM08-0839	Australia: Queensland	BOLD IDS
C. gangis	gang4	IMLS124-12	IM11-0125	Australia: Queensland	BOLD IDS
C. gangis	gang5	LOQ003-04	04HBL004003	Australia: Queensland	BOLD IDS
C. gangis	gang5	LOQ006-04	04HBL004006	Australia: Queensland	BOLD IDS
C. gangis	gang6	LOQ004-04	04HBL004004	Australia: Queensland	BOLD IDS
C. gangis	gang7	LOQ005-04	04HBL004005	Australia: Queensland	BOLD IDS
C. gangis	gang8	LOQ007-04	04HBL004007	Australia: Queensland	BOLD IDS
C. gangis	gang9	LOQ008-04	04HBL004008	Australia: Queensland	BOLD IDS
C. gangis	gang10	LOQC058-05	05-QLD-00058	Australia: Queensland	BOLD IDS
C. gangis	gang10	LOQC059-05	05-QLD-00059	Australia: Queensland	BOLD IDS
C. gangis	gang10	LOQTI489-11	gvc16644-1L	Australia: Queensland	BOLD IDS
C. gangis	gang10	LOQTI501-11	gvc16669-1L	Australia: Queensland	BOLD IDS
C. leucanioides	leuc1	HQ682744	JDF0013	Yemen: Ibb, Lower Wadi Duur	[34]
C. leucanioides	leuc2	HQ682743	JDF0014	Yemen: Taizz, Wadi, Warzan	[34]
C. leucanioides	leuc3	HQ682742	JDF0015	Yemen: Ibb, Lower Wadi Duur	[34]
C. leucanioides	leuc4	HQ682741	JDF0016	Yemen: Taizz, Wadi, Warzan	[34]
C. leucanioides	leuc5	HQ682740	JDF0017	Yemen: Lahij Governorate, Al Dhala	[34]
C. leucanioides	leuc6	HQ682739	JDF0018	Yemen: al-Hudaydah, Jabal Bura foothills	[34]
C. leucanioides	leuc7	HQ682738	JDF0019	Yemen: Al-Mahwit	[34]
C. leucanioides	leuc8	HQ682746	JDF0020	Kenya: Eastern Kibwezi	[34]
C. leucanioides	leuc8	HQ682745	JDF0021	Kenya: Eastern Kibwezi	[34]
C. transiens	tran7	KJ380885	M48	India: Western Ghats	GenBank
C. transiens	tran8	KP233790	RO_HP2	India: Chamba, Himachal Pradesh	GenBank
C. transiens	tran9	PMANL3219-14	USNM ENT 00980900	Malaysia: Sabah (Borneo)	BOLD IDS
C. transiens	tran12	LPMLY227-14	BIOUG12316-D01	Malaysia: Sabah (Borneo)	BOLD IDS
C. transiens	tran17	PMANL3218-14	USNM ENT 00980899	Malaysia: Sabah (Borneo)	BOLD IDS
C. transiens	tran18	LEPMY1052-14	BIOUG13574-G07	Malaysia: Pahang	BOLD IDS
C. transiens	tran19	LEPMY1057-14	BIOUG13574-G12	Malaysia: Pahang	BOLD IDS
C. transiens	tran10	QMA2321-13	ARB00024603	China: Yunnan	BOLD IDS
C. transiens	tran10	QMA2322-13	ARB00024604	China: Yunnan	BOLD IDS

Species	Haplotype Code	GenBank / BOLD IDS acc. no.	Voucher no.	Locality	Data source***
C. transiens	tran11	QMA2323-13	ARB00024605	China: Yunnan	BOLD IDS
C. transiens	tran11	QMA2324-13	ARB00024606	China: Yunnan	BOLD IDS
C. transiens	tran10	QMA2325-13	ARB00024607	China: Yunnan	BOLD IDS
C. transiens	tran13	GU696198	AYK-04-0776-16	China: Taiwan	GenBank
C. transiens	tran14	KX861984	NIBGE MOT-01750	Pakistan: Islamabad	GenBank
C. transiens	tran15	KX863160	NIBGE MOT-01751	Pakistan: Kashmir	GenBank
C. transiens	tran16	KX861774	NIBGE MOT-02900	Pakistan: Khyber Pakhtunkhwa	GenBank
C. transiens	tran14	JF858058	NIBGE MOT-00869	Pakistan: Azad Kashmir	GenBank
C. transiens	tran14	JF858059	NIBGE MOT-00870	Pakistan: Azad Kashmir	GenBank
C. transiens	tran15	JF858060	NIBGE MOT-00871	Pakistan: Azad Kashmir	GenBank
C. transiens	tran1	KY683813	Sph615	Laos: Phongsali Province	Present study
C. transiens	tran2	KY683814	Sph618	Thailand: Loei Province	Present study
C. transiens	tran2	KY683815	Sph619	Thailand: Loei Province	Present study
C. transiens	tran2	KY683816	Sph621	Thailand: Loei Province	Present study
C. transiens	tran2	KY683817	Sph622	Thailand: Maehongson	Present study
C. transiens	tran2	KY683818	Sph626	Thailand: Loei Province	Present study
C. transiens	tran1	KY683819	Sph627	Thailand: Maehongson	Present study
C. transiens	tran3	KY683820	Sph640	Myanmar: Kachin State	Present study
C. transiens	tran2	KY683822	Sph667	Myanmar: Mandalay Region	Present study
C. transiens	tran1	KY683823	Vie1	Vietnam: Thanh Hoa Province	Present study
C. transiens	tran2	KY683824	Vie2	Vietnam: Thanh Hoa Province	Present study
C. transiens	tran1	KY683825	Vie3	Vietnam: Thanh Hoa Province	Present study
C. transiens	tran2	KY683826	Vie4	Vietnam: Thanh Hoa Province	Present study
C. transiens	tran2	KY683827	Vie5	Vietnam: Thanh Hoa Province	Present study
C. transiens	tran5	KY683828	Vie6	Vietnam: Vinh Phuc Province	Present study
C. transiens	tran1	KY683829	Vie7	Vietnam: Vinh Phuc Province	Present study
C. transiens	tran6	KY683830	Vie8	Vietnam: Vinh Phuc Province	Present study
C. transiens	tran1	KY683831	Vie9	Vietnam: Vinh Phuc Province	Present study
C. transiens	tran2	KY683832	Vie10	Vietnam: Vinh Phuc Province	Present study
C. transiens	tran4	KY683821	Sph641	Myanmar: Kachin State	Present study
*Arctia menetriesii***	n/a	KM111174	IEPN-G0703	Russia: Arkhangelsk Region	Present study
*Arctia tundrana***	n/a	KM111175	IEPN-G0704	Russia: Arkhangelsk Region	[43]

*Populations of *C. gangis*: (i) Arabia and South Asia: Oman + Pakistan + India + Nepal; (ii) mainland Southeast Asia: Myanmar + Vietnam + Thailand + South China; (iii) Australia; and (iv) Lesser Sundas. **An outgroup. ***We used only published sequences from BOLD IDS and GenBank that are freely available to any researcher.
