# Supplementary material for: DNA barcoding unravels contrasting evolutionary history of two widespread Asian tiger moth species during the Late Pleistocene
Source: PLoS One. 2018 Apr 4;13(4):e0194200. doi: 10.1371/journal.pone.0194200 (PMC5884489; doi:10.1371/journal.pone.0194200)
Supplement: S2 Table — (PDF) [file pone.0194200.s004.pdf]

S2 Table. Genetic divergences (mean uncorrected *p*-distance ± standard error estimations, %) between populations and taxa of *Creatonotos* spp. based on the mitochondrial COI gene fragment*

**Populations and taxa**	***C. gangis*, Arabia and South Asia (*N* = 20)**	***C. gangis*, mainland Southeast Asia (*N* = 20)**	***C. gangis*, Lesser Sundas (*N* = 2)**	***C. gangis*, Australia (*N* = 15)**	***C. transiens* (*N* = 39)**
C. gangis, mainland Southeast Asia (*N* = 20)	1.78±0.45				
C. gangis, Lesser Sundas (*N* = 2)	1.58±0.50	1.58±0.43			
C. gangis, Australia (*N* = 15)	1.61±0.52	1.18±0.31	1.29±0.48		
C. transiens (*N* = 39)	6.91±1.16	6.41±1.11	6.72±1.19	6.43±1.16	
C. leucanioides (*N* = 3)	8.03±1.23	7.94±1.21	8.56±1.29	7.96±1.24	6.65±1.17

*Arabia and South Asia: Oman (*C. omanirana*) + Pakistan + India + Nepal; and mainland Southeast Asia: Myanmar + Vietnam + Thailand + South China.
