# Supplementary material for: DNA barcoding unravels contrasting evolutionary history of two widespread Asian tiger moth species during the Late Pleistocene
Source: PLoS One. 2018 Apr 4;13(4):e0194200. doi: 10.1371/journal.pone.0194200 (PMC5884489; doi:10.1371/journal.pone.0194200)
Supplement: S3 Table — (PDF) [file pone.0194200.s005.pdf]

S3 Table. Molecular diversity indexes and population expansion test statistics of *Creatonotos* spp. samples based on the COI sequences

Parameters	*C. gangis**					*C. transiens*, Eurasia
	Arabia and South Asia	Mainland Southeast Asia	Eurasia	Australia	All available sequences	
Sample size, *N*	20	20	40	15	57	39
No. of haplotypes	6	11	16	4	22	
Haplotype diversity (h±SD)	0.658±0.096	0.868±0.064	0.876±0.034	0.543±0.133	0.909±0.020	0.854±0.040
Nucleotide diversity (π±SD), %	0.4±0.3	1.1±0.6	1.3±0.7	0.2±0.2	1.3±0.7	0.6±0.3
Fu's F_S-test:						
F_S	-0.629	-0.704	-1.925	-0.626	-4.763	-7.59565
P-value	0.364	0.278	0.249	0.274	0.066	<0.00001
Tajima's D-test:						
D	-0.743	-0.095	0.281	-0.922	-0.156	-1.769
P-value	0.274	0.501	0.687	0.221	0.526	0.017
Mismatch analysis (spatial expansion model):						
Estimated τ	2.265	6.595	5.241	0.236	6.127	0.694
95% confidence interval	0.518-4.354	3.378-9.912	3.059-11.333	0.071-3.478	3.948-9.004	0.515-5.922
Model (SSD) *P*-value	0.273	0.331	0.317	0.736	0.557	0.596
*Time since population expansion inferred from parameter τ, ka:***						
Mean	639.8	1863.0	1480.5	66.7	1730.8	196.0
95% confidence interval	146.3-1229.9	954.2-2800.0	864.1-3201.4	20.1-982.5	1115.3-2543.5	145.5-1672.9

*Arabia and South Asia: Oman + Pakistan + India + Nepal; mainland Southeast Asia: Myanmar + Vietnam + Thailand + South China; and all available sequences: Eurasia + Lesser Sundas + Australia. **The 'mid-Aegean' insect mutation rate = 1.77×10^{-8} s/s/y [66].
