# Supplementary material for: DNA barcoding unravels contrasting evolutionary history of two widespread Asian tiger moth species during the Late Pleistocene
Source: PLoS One. 2018 Apr 4;13(4):e0194200. doi: 10.1371/journal.pone.0194200 (PMC5884489; doi:10.1371/journal.pone.0194200)
Supplement: S4 Table — (PDF) [file pone.0194200.s006.pdf]

S4 Table. Model parameters estimated from posterior distribution of the scenario 2-CG concerning origin of *Creatonotos gangis* populations within the ABC framework

**Parameters**	**Mean**	**Median**	**qt 5%**	**qt 95%**
Effective population size				
Southeast Asian population (N_1)	1.21×10^6	9.78×10^5	2.84×10^5	2.96×10^6
Arabian – South Asian population (N_2)	1.04×10^6	7.38×10^5	1.76×10^5	3.05×10^6
Hypothetical founder population (N_{2b})	5.12×10^3	2.63×10^5	6.80×10^2	9.50×10^3
Australian population (N_3)	2.65×10^5	4.20×10^3	6.10×10^4	4.74×10^5
Hypothetical founder population (N_{3b})	4.51×10^3	5.14×10^3	5.94×10^2	9.29×10^3
Divergence time estimation				
Time of split between Southeast Asian and Arabian – South Asian populations (t_2), ka	143.0	109.0	55.8	359.0
Time of split between Southeast Asian and Australian populations (t_1), ka	62.6	65.1	19.3	96.9
Period of low effective population size N_{3b} since the colonization of Australia (t_{cb}), ka	2.8	2.8	0.8	4.7
Mutation rate inferred from the mitochondrial COI gene				
μ_{ABC}, s/s/y	1.52×10^{-8}	1.35×10^{-8}	1.03×10^{-8}	2.58×10^{-8}
