# Supplementary material for: DNA barcoding unravels contrasting evolutionary history of two widespread Asian tiger moth species during the Late Pleistocene
Source: PLoS One. 2018 Apr 4;13(4):e0194200. doi: 10.1371/journal.pone.0194200 (PMC5884489; doi:10.1371/journal.pone.0194200)
Supplement: S5 Table — (PDF) [file pone.0194200.s007.pdf]

S5 Table. Prior assumptions and settings of the two biogeographical scenarios of the origin of *Creatonotos gangis* populations, which were tested under an ABC framework*

<table>
<tr><th>Scenarios</th><th>Basic assumptions</th><th>Time frames</th><th>Prior setting of divergence time intervals, years</th><th>Prior setting of effective population size and mutation model</th></tr>
<tr><td>Scenario 1-CG</td><td>Arabian–South Asian and Australian populations independently derived from Southeast Asian population</td><td>Split between Arabian–South Asian and Southeast Asian populations was before the mid-Pleistocene; and Australian population derived before the Late Pleistocene</td><td>Uniform:
$t_{2a} = 5\times10^5$-5×10^6
$t_{1a} = 10^5$-10^6
$t\text{-}db = 5\times10^2$-$5\times10^3$</td><td rowspan="2">Effective population size:
Uniform:
$N_1 = 5\times10^4$-5×10^6
$N_2 = 5\times10^4$-5×10^6
$N_3 = 5\times10^3$-5×10^5
$N_{2b} = 10^2$-10^4
$N_{3b} = 10^2$-10^4

Evolutionary model:
HKY+G**

Mutation rate:
Uniform:
$\mu = 1\times10^{-8}$-1×10^{-6} s/s/y***</td></tr>
<tr><td>Scenario 2-CG</td><td>Same assumption</td><td>Split between Arabian–South Asian and Southeast Asian populations was since the mid-Pleistocene; and Australian population derived during the Late Pleistocene</td><td>Uniform:
$t_2 = 5\times10^4$-5×10^5
$t_1 = 10^4$-10^5
$t\text{-}db = 5\times10^2$-$5\times10^3$</td></tr>
</table>

*Southeast Asian population: samples from Myanmar, Vietnam, Thailand, and South China, Arabian–South Asian population: samples from Oman, Pakistan, India, and Nepal, and Australian population. **Based on the AICc of MEGA6 [52]. ***The rate range is given with respect to the following works: Papadopoulou et al. [66], and Molak and Ho [71]. Explanation of codes of time intervals and effective population size parameters are presented in the caption to Fig. 4.
